# ICE*Sag37*, a Novel Integrative and Conjugative Element Carrying Antimicrobial Resistance Genes and Potential Virulence Factors in *Streptococcus agalactiae*

**DOI:** 10.3389/fmicb.2017.01921

**Published:** 2017-10-05

**Authors:** Kaixin Zhou, Lianyan Xie, Lizhong Han, Xiaokui Guo, Yong Wang, Jingyong Sun

**Affiliations:** ^1^Department of Clinical Microbiology, Ruijin Hospital, Shanghai Jiao Tong University School of Medicine, Shanghai, China; ^2^Department of Medical Microbiology and Parasitology, Institutes of Medical Sciences, Shanghai Jiao Tong University School of Medicine, Shanghai, China; ^3^Department of Laboratory Medicine, Shandong Provincial Hospital, Shandong University, Jinan, China

**Keywords:** *Streptococcus agalactiae*, MDR, integrative and conjugative element (ICE), virulence, ICESa2603 family

## Abstract

ICE*Sag37*, a novel integrative and conjugative element carrying multidrug resistance and potential virulence factors, was characterized in a clinical isolate of *Streptococcus agalactiae*. Two clinical strains of *S. agalactiae*, Sag37 and Sag158, were isolated from blood samples of new-borns with bacteremia. Sag37 was highly resistant to erythromycin and tetracycline, and susceptible to levofloxacin and penicillin, while Sag158 was resistant to tetracycline and levofloxacin, and susceptible to erythromycin. Transfer experiments were performed and selection was carried out with suitable antibiotic concentrations. Through mating experiments, the erythromycin resistance gene was found to be transferable from Sag37 to Sag158. *Sma*I-PFGE revealed a new *Sma*I fragment, confirming the transfer of the fragment containing the erythromycin resistance gene. Whole genome sequencing and sequence analysis revealed a mobile element, ICE*Sag37*, which was characterized using several molecular methods and *in silico* analyses. ICE*Sag37* was excised to generate a covalent circular intermediate, which was transferable to *S. agalactiae*. Inverse PCR was performed to detect the circular form. A serine family integrase mediated its chromosomal integration into *rumA*, which is a known hotspot for the integration of streptococcal ICEs. The integration site was confirmed using PCR. ICE*Sag37* carried genes for resistance to multiple antibiotics, including erythromycin [*erm(B)*], tetracycline [*tet(O)*], and aminoglycosides [*aadE, aphA*, and *ant(6)*]. Potential virulence factors, including a two-component signal transduction system (*nisK/nisR*), were also observed in ICE*Sag37*. S1-PFGE analysis ruled out the existence of plasmids. ICE*Sag37* is the first ICE*Sa2603* family-like element identified in *S. agalactiae* carrying both resistance and potential virulence determinants. It might act as a vehicle for the dissemination of multidrug resistance and pathogenicity among *S. agalactiae*.

## Introduction

*Streptococcus agalactiae* (group B streptococcus) is the leading cause of neonatal sepsis and meningitis in many countries. It is also an important pathogen among pregnant women, immunocompromised adults, and the elderly (Le Doare and Heath, 2013).

Erythromycin resistance in *S. agalactiae* has increased in the last decade in several countries, with slight geographical variations (Betriu et al., 2003). A recent study had reported that the prevalence of erythromycin resistance in *S. agalactiae* was 61.5% in Shanghai, China, and 69.8% of this was associated with the gene *ermB* (Yan et al., 2016). Macrolide resistance is attributed to two principal mechanisms, a methylase-mediated target site modification encoded by the *erm* genes, and an active efflux mediated by the *mef* genes (Leclercq, 2002; Varaldo et al., 2009). The *erm* genes modify the post-transcriptional methylation of an adenine residue in the 23S rRNA of bacterial ribosomes and render co-resistance to macrolides, lincosamides, and streptogramins B antibiotics (MLS_B_), both constitutive (cMLS_B_) and inducible (iMLS_B_) ones (Dzyubak and Yap, 2016; Mingoia et al., 2016). Insertions, duplications, deletions, and missense mutations within the leader regulatory regions upstream of the *erm* gene are commonly found in the constitutively expressed *erm* operons (Dzyubak and Yap, 2016). High rates of tetracycline resistance have also been observed in *Streptococcus* species (Luna and Roberts, 1998; Chopra and Roberts, 2001; Princivalli et al., 2009; Cattoir, 2016). Both *tet(M)* and *tet(O)* encode tetracycline resistance ribosomal protection proteins (RPRs). *tet(M)* is considered the most prevalent, due to its association with the broad host range Tn*916*/Tn*1545* family of conjugative transposons (Roberts and Mullany, 2011; Warburton et al., 2016). *tet(O)* has been discovered chromosomally in several Gram-positive organisms such as *Streptococcus* and *Staphylococcus* species (Nguyen et al., 2014).

Integrative and conjugative elements (ICEs) comprise a diverse group of mobile genetic elements found in both Gram-positive and Gram-negative bacteria. They reside in the host chromosome, but retain the ability to excise and transfer through conjugation (Wozniak and Waldor, 2010). ICEs employ integrases to integrate into the host chromosome and a type IV secretion system is always encoded to mediate the conjugation (Johnson and Grossman, 2015). They bestow a wide range of phenotypes on their hosts through the cargo genes they carry, such as antibiotic resistance determinants, virulence factors, and metabolic genes (Wozniak and Waldor, 2010; Johnson and Grossman, 2015). The transfer of ICEs enhances the fitness of microbes in their hosts and exerts a major effect on bacterial evolution and adaption. So far, several ICEs have been reported in *S. agalactiae*, including ICE*Sa2603* and ICE*Sag2603VR-1* in *S. agalactiae* 2603V/R (Tettelin et al., 2002) and Tn*GBS1* and Tn*GBS2* in *S. agalactiae* NEM316 (Glaser et al., 2002).

In this study, we described the molecular characterization of a novel ∼75-kb *erm(B)*-carrying ICE, ICE*Sag37*, which has never before been reported in *S. agalactiae*.

## Materials and Methods

### Bacterial Strains and Antimicrobial Susceptibility Test

A clinical strain of *S. agalactiae*, Sag37, was isolated from the blood sample of a new-born with bacteremia in Shanghai in 2014. Another clinical strain of *S. agalactiae*, Sag158, was isolated from the blood sample of another new-born, and was used as the recipient. The minimum inhibitory concentrations (MICs) of erythromycin, tetracycline, levofloxacin, clindamycin, and penicillin were determined using the Etest (bioMe’rieux, France), and the results were interpreted based on the guidelines of the Clinical and Laboratory Standards Institutes [CLSI] (2014).

### Multilocus Sequence Typing Analysis (MLST) and Serotyping

Multilocus sequence typing analysis (MLST) and serotyping was performed as previously described (Jones et al., 2003; Imperi et al., 2010). After MLST, an online database^1^ was used for assigning allele numbers, and the sequence types (STs) were obtained from the combination of the results.

### Conjugal Transfer Experiments, Pulsed-Field Gel Electrophoresis (PFGE), and S1-Nuclease Pulsed-Field Gel Electrophoresis (S1-PFGE)

Conjugal transfer experiments were performed in broth culture using the strain Sag37 (erythromycin-resistant and levofloxacin-susceptible) as the donor and the strain Sag158 (erythromycin-susceptible and levofloxacin-resistant) as the recipient. Briefly, donor and recipient strains were grown to an OD_600_ of 0.4 and mixed in a ratio of 1:1 in 2 ml of culture. The suspension was pelleted, resuspended, and used for mating. Selection was performed with suitable concentrations of erythromycin (1 mg/L) and levofloxacin (8 mg/L). All transfer experiments were performed at least thrice. Sag158 and the transconjugant, Sag158-1, were both digested with *Sma*I endonuclease and S1 nuclease and subjected to PFGE and S1-PFGE, as described in previous studies (Barton et al., 1995; Elliott et al., 1998). Briefly, two or three colonies were selected from a fresh plate of overnight growth and incubated in Todd-Hewitt broth for 5 h at 35°C; this suspension was centrifuged and the pellet was re-suspended in Tris–NaCl buffer. The agarose-bacterium plugs were generated and incubated overnight at 35°C in lysis solution. The plugs were washed several times and digested with a suitable nuclease. The chromosomal digests were separated by PFGE. Pulsotypes were clustered based on a cut-off of 70% similarity.

### DNA Sequencing and Sequence Analysis

The genomic DNA was extracted using the QIAGEN Midi Kit (Qiagen, Hilden, Germany). The DNA of Sag37 and Sag158 was sequenced using the pacbio RS II (Pacific Biosciences, Menlo Park, CA, United States). The reads were *de novo* assembled using the HGAP 3.0 SMRT^TM^ Pipe. The transconjugant was sequenced using the illumina HiSeq sequencing approach. SOAP *de novo*^2^ was used to perform genome assembly. Sequence similarity searches were carried out using BLAST^3^. Open reading frames (ORFs) were predicted using the ORF Finder software^4^. Protein-coding genes were initially identified and annotated using RAST. The antimicrobial resistance genes were identified using ResFinder^5^.

### Amplification Experiments

**FIGURE 1:**
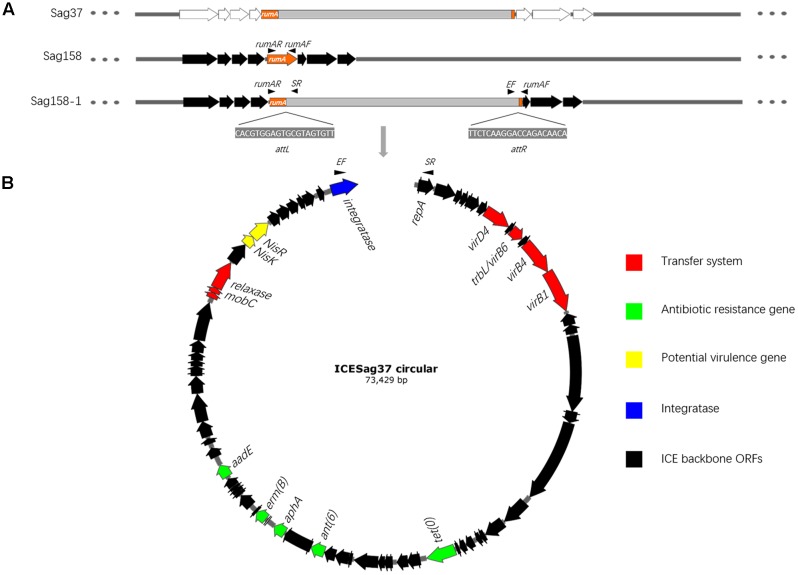
**(A)** Gray and orange fragments represent ICE*Sag37* and the *rumA* gene, respectively. The surrounding genes are colored white and black. The site-specific integration of ICE*Sag37* into the *rumA* gene is clearly visible. The 20-bp attL and attR sequences of ICE*Sag37* and primers, rumAF and rumAR, are also shown. **(B)** Schematic diagram of the circular form of ICE*Sag37*. Black arrows indicate the positions and directions of outward-directed primers, SR and EF. The core genes are shown in the circular form of ICE*Sag37*. Color codes are defined in the inset.

PCR assays were performed with three pairs of primers (**Table 1**), rumAF and SR; rumAR and EF; and rumAF and rumAR (**Figure 1**), to confirm its site-specific integration: *rumA*. Outward-directed primers (SR and EF) were used to detect the circular form of ICE*Sag37*. DNA preparation, amplification, and electrophoresis of the PCR products were carried out by previously established procedures (Hynes et al., 1992) in conditions indicated for the use of individual primer pairs. The PCR products were verified by sequencing.

**Table 1:** Principal oligonucleotide primers used in this study.

Gene	Primer	Sequence(5′-3′)	Product size (bp)
***int***	intF	GCAACGTGGTGAATTGATAGGG	1011
	intR	AAAACTGCACGATCAAACTCCG	
***rumA***	rumAF	CAGGTACCAGTTCGTCGTGT	1092
	rumAR	CGCAGTCGACATCCAAACAG	
***repA***	SR	GTCGCACATACGAAACAGCG	
***Int***	EF	TTCGTGTGTCACTGGAACCG	

### Nucleotide Sequence Accession Numbers

The nucleotide sequences of Sag37 and Sag158 have been deposited in the Genbank sequence database under the accession numbers CP019978 and CP019979, respectively. The sequence read archive (SRA) of Sag158-1 has been deposited in Genbank under the accession number SRR5585701.

## Results

### Early Characterization of Sag37 and Sag158

Sag37 was highly resistant to erythromycin, clindamycin, and tetracycline and susceptible to levofloxacin and penicillin, while Sag158 was resistant to tetracycline and levofloxacin, and susceptible to erythromycin, clindamycin, and penicillin. Sag37 and Sag158 displayed different STs (12 and 19, respectively) and serotypes (Ib and III, respectively). All these results, the isolation data, and the resistance genes of the two strains are summarized in **Table 2**.

**Table 2:** Relevant features of the two strains and the transconjugant of *Streptococcus agalactiae.*

Strain	Source	Serotype	ST	MIC(mg/L)					Resistance genes
				*E*	TET	LEV	CM	*P*	
Sag37	Blood	Ib	12	>32	12	0.50	>32	0.032	*erm(B), tet(O), aadE, aphA, ant(6)*
Sag158	Blood	III	19	0.125	16	>32	0.094	0.032	*tet(M)*
Sag158-1	/	III	19	>32	24	>32	>32	0.032	*erm(B), tet(O), tet(M), aadE, aphA, ant(6)*

*E, erythromycin; TET, tetracycline; LEV, levofloxacin; CM, clindamycin; P, penicillin*.

### Transfer of Erythromycin Resistance

**FIGURE 2:**
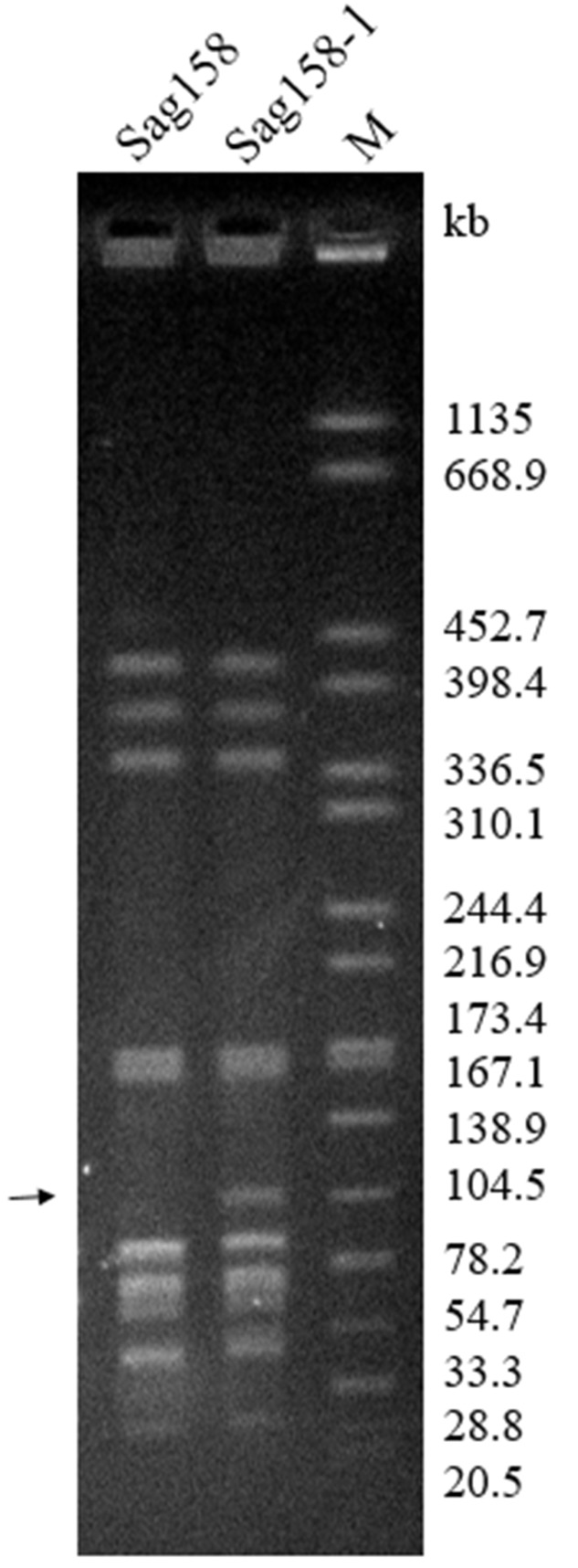
Pulsed-field gel electrophoresis (PFGE) profiles of *S. agalactiae* Sag158 and its transconjugant, Sag158-1. PFGE analysis showed the appearance of a new *Sma*I fragment (black arrow). M, *Salmonella enterica* serotype Braenderup H9812 was digested with *Xba*I and used as a molecular size marker.

Through mating experiments, erythromycin resistance was transferred from the donor, Sag37 (erythromycin-resistant and levofloxacin-susceptible), to the recipient, Sag158 (erythromycin-susceptible and levofloxacin-resistant), at a frequency of ∼3 × 10^-7^. Randomly selected erythromycin-resistant transconjugants were used for further experiments. The principle features and MICs of the transconjugant, Sag158-1, are summarized in **Table 1**. Sag158-1 showed the same erythromycin MIC as the donor and a higher tetracycline MIC than the donor strain. *SmaI*-PFGE analysis indicated a new *SmaI* fragment (**Figure 2**). S1-PFGE revealed that Sag37 harbored no plasmids. The erythromycin resistance was also transferred from Sag37 to two other clinical *S. agalactiae* recipients, although the data is not shown in this paper.

### General Genomic Features and Detection of the Mobile Element

Whole genome sequencing was carried out for the two strains investigated in this study, Sag37 and Sag158. The genomes of Sag37 and Sag158 comprised a single circular chromosome and were 2,198,785 and 2,096,882 bp in length, respectively, with average G + C contents of 35.77 and 35.66%, respectively. A total of 2180 protein coding sequences (CDS) were predicted in Sag37, while 2093 CDS were predicted in Sag158. Different substitutions were found in the quinolone resistance-determining regions of Sag158 (Ser81Leu in *gyrA* and Ser79Tyr and Ser140Pro in *parC*), which were not found in Sag37.

In order to track possible genomic clues regarding the mobile elements, comparative genome analyses were performed for Sag37, Sag158, and Sag158-1. We identified a contig in Sag158-1, which contained all the resistance genes that were present in Sag37, but were absent in Sag158. We aligned this contig with Sag37 and Sag 158, and confirmed that it did exist in Sag37, but was absent in Sag158. Further sequence analysis confirmed the presence of a ∼75-kb mobile element carrying *erm(B)* and *tet(O)*.

### Circularization and Integration Site of ICE*Sag37*

PCR assays using outward-directed primers (SR and EF) revealed that the mobile element could excise and form a covalently circular intermediate in both Sag37 and Sag158-1 (**Figure 1**). Sequence analysis of the amplicons and the left and right junctions of the mobile element enabled us to identify the putative core sites (attI, attL, and attR) and obtain the complete sequence.

Sequences adjacent to the ends of the mobile element in Sag37 and Sag158-1 suggested a possible integration site. PCR assays with three pairs of primers, rumAF and SR; rumAR and EF; and rumAF and rumAR (**Figure 1**), and sequence analysis of the amplicons confirmed the site-specific integration. The mobile element encoded a serine family integrase, which mediated chromosomal integration at the 3′ end of the conserved gene, *rumA* (23S rRNA [uracil-5-]-methyltransferase). The integrase was also confirmed through a PCR assay.

### Characterization of ICE*Sag37*

**FIGURE 3:**
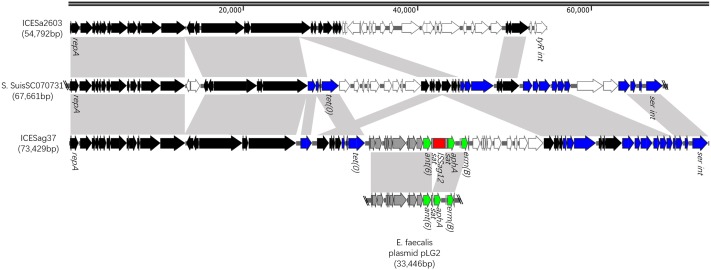
Schematic, but to scale, representation of ICE*Sag37* from *S. agalactiae* Sag37 and linear DNA comparison against ICE*Sa2603* and fragments of *S. suis* SC070731 an*E. faecalis* plasmid. The 30 conserved core genes of the ICE*Sa2603* family backbone are indicated by black arrows. Genes homologous to *S. suis* SC070731 and *E. faecalis* plasmid are indicated by blue and gray arrows, respectively. Homologous regions are shaded in gray.

The results of the mating experiments, the PFGE, and the PCR assays provided a glimpse of the mobile element. The results of the whole genome sequencing and the extensive sequence analysis revealed its full picture. It was designated ICE*Sag37* and is schematically represented in **Figure 3**. ICE*Sag37* was found to be 73,429 kb in size (629058–702486 bp in Sag37) and predicted to contain 79 ORFs. In addition to the serine family integrase, ICE*Sag37* also contained several genes belong to a type IV secretion system (T4SS), as well as genes encoding putative mobilization proteins of the *MobC* family and relaxases (**Figure 1**). The transferability of ICE*Sag37*, as shown in the conjugation experiments, strongly suggested that it had an intact and feasible transfer system.

ICE*Sag37* carried resistance genes to multiple antibiotics, including erythromycin [*erm(B)*], tetracycline [*tet(O)*], and aminoglycosides [*aadE, aphA*, and *ant(6)*]. The mosaic genes from the *Enterococcus faecalis* plasmid, pLG2, were also observed in ICE*Sag37* (**Figure 2**), and it was this plasmid that introduced the erythromycin resistance determinant, *erm(B)*, as well as the aminoglycoside resistance gene cluster containing *ant(6)* and *aphA*, into ICE*Sag37*. IS*Sag12* was integrated into the mosaic genes, disrupting the sat gene. *tet(O)* and *aadE* were also embedded in ICE*Sag37*, although the source was not known. In addition to the antibiotic resistance genes, ICE*Sag37* also contained genes predicted to be involved in virulence, a two-component signal transduction system (*nisK/nisR)*, from *S. suis.*

## Discussion

A recent study had shown that *erm(B)* accounts for the vast majority of erythromycin resistance in *S. agalactiae* in China, with the most predominant phenotype being cMLS_B_ (77.5%) (Yan et al., 2016). The strain used in this study, *S. agalactiae* Sag37, also constitutively expressed MLS_B_ resistance. The leader peptide in ICE*Sag37* contained a duplicated TAAA motif, generating an earlier stop codon and resulting in a leader peptide that was 9 amino acids shorter (27 amino acids). Various point mutations were also observed in the leading peptide, including substitutions of ATT for GTT and GTA for GCA at leader codons 15 and 25, respectively. However, these changes were observed in both inducible and constitutive strains (Oh et al., 1998; Rosato et al., 1999; Min et al., 2008; Feld et al., 2009); therefore, a firm conclusion cannot be stated and further investigation will be required to explain the mechanism.

ICE*Sag37* was discovered in the strain *S. agalactiae* Sag37 in this study. Interestingly, homology searches of the ICE*Sag37* sequence showed that it had the highest similarity to *S. suis* SC070731 (Genbank No. CP003922), but also shared its backbone with ICE*Sa2603*. ICE*Sa2603*, a ∼54-kb ICE first discovered in *S. agalactiae* 2603V/R (Tettelin et al., 2002), is considered the prototype of the ICE family, based on the integrases (Bi et al., 2012). ICE*Sag37* contained all 30 conserved core genes present in the ICE*Sa2603* family backbone (Huang et al., 2016), but encoded a serine family integrase instead of *int_ICESa2603_*, a tyrosine family integrase. It is to be noted that a known hypervariable region in ICE*Sa2603* (white arrows in **Figure 2**) (Huang et al., 2016) corresponded to the region where we found the mosaic genes in ICE*Sag37*. Thus, we referred to ICE*Sag37* as ICE*Sa2603* family-like ICE. The serine family integrase it encoded mediated its chromosomal integration into the 3′ end of the conserved gene, *rumA* (23S rRNA [uracil-5-]-methyltransferase), which is a known hotspot for the integration of streptococcal ICEs (Ambroset et al., 2016).

Although we do not have sufficient data to pinpoint the origins and assembly of ICE*Sag37*, it was apparent that mobilization and recombination events played key roles in its assembly. ICE*Sag37* comprised genes from both phylogenetically close and distant species of *Streptococcus*. Notably, the antibiotic resistance gene cluster in ICE*Sag37* was homologous to a plasmid in *E. faecalis*, indicating that inter-species genetic exchange also occurred, thus broadening the host range and dissemination of combined cargo genes. Although the sources of *tet(O)* and *aadE* were not clear, genes involved in mobility, including the *TnpV* and recombinase genes, were found surrounding them. Thus, we proposed that the two resistance genes also originated from mobile elements. However, further research is needed to characterize the mechanisms behind the transfer and recombination of ICEs. Whole-genome comparisons revealed the diversity of the flexible gene pool of *S. agalactiae*, suggesting the open nature of its pan-genome (Tettelin et al., 2002; Brochet et al., 2008). This result indicated that the genome microevolution of pathogens should be viewed from a different perspective, as the ICE pool may be communal.

Although many ICEs have been characterized in *Streptococcus* species, to the best of our knowledge, ICE*Sag37* is the first ICE*Sa2603* family-like element carrying both resistance and potential virulence determinants identified in *S. agalactiae*. Many ICEs containing both resistance and virulence determinants have been reported in *S. suis* isolated from diseased pigs or humans, such as ICE*Ssu32457*, ICE*Sluvan*, and ICE*Ssu (BM407)2* (Holden et al., 2009; Palmieri et al., 2012; Bjorkeng et al., 2013). *S. suis* infection is notorious for causing serious zoonotic diseases and for causing large-scale outbreaks of STSS in China (Chen et al., 2007). Remarkably, ICE*Sag37* showed a striking similarity to *S. suis* SC070731 and contained its two-component signal transduction system, *nisK/nisR*, which has been proven to contribute to the virulence of *S. suis in vitro* and *in vivo* (Xu et al., 2014). Thus, we proposed that the novel ICE might be a vehicle for the dissemination of not only multidrug resistance, but also potential pathogenicity, among *S. agalactiae* and that extensive screening for ICE*Sag37* is required.

## Author Contributions

All authors listed have made a substantial, direct and intellectual contribution to the work, and approved it for publication.

## Conflict of Interest Statement

The authors declare that the research was conducted in the absence of any commercial or financial relationships that could be construed as a potential conflict of interest.

## Footnotes

1 http://pubmlst.org/sagalatiae

2 http://soap.genomics.org.cn/

3 http://www.ncbi.nlm.nih.gov

4 http://ncbi.nlm.nih.gov/gorf/gorf.html

5 https://cge.cbs.dtu.dk/services/ResFinder/
